# Circulating MicroRNAs as Potential Diagnostic Biomarkers for Diabetic Retinopathy: A Meta-Analysis

**DOI:** 10.3389/fendo.2022.929924

**Published:** 2022-07-08

**Authors:** Lingli Ma, Yan Wen, Zimeng Li, Nan Wu, Qing Wang

**Affiliations:** ^1^ Department of Endocrinology and Metabolism, China-Japan Union Hospital of Jilin University, Changchun, China; ^2^ Department of Dermatology, China-Japan Union Hospital of Jilin University, Changchun, China

**Keywords:** miRNA, diabetic retinopathy, diagnosis, biomarkers, meta-analysis

## Abstract

**Objective:**

Diabetic retinopathy (DR) is a common diabetic microvascular complication and a major cause of acquired vision loss. Finding effective biomarkers for the early identification and diagnosis of DR is crucial. This study aimed to comprehensively evaluate the accuracy of microRNAs (miRNAs) in the diagnosis of DR *via* a meta-analysis of previously published diagnostic studies. This study has been registered on the PROSPERO website, with the number CRD42022323238.

**Methods:**

We searched PubMed, Cochrane Library, Embase, Web of Science, China Wanfang database, and China Knowledge Network database to identify relevant articles published from the time of database creation to April 10, 2022. Stata 14.0 software was used to calculate the pooled sensitivity, specificity, positive likelihood ratio (PLR), negative likelihood ratio (NLR), diagnostic ratio (DOR), and area under the summary receiver operating characteristic (ROC) curve to assess the accuracy of miRNAs in the diagnosis of DR. Heterogeneity between studies was assessed using Cochran-Q test and I^2^ statistic for quantitative analysis. The random-effect model was selected due to significant heterogeneity. Subgroup analysis and regression analysis were also performed to determine the potential sources of heterogeneity.

**Results:**

We included 25 articles detailing 52 studies with 1987 patients with DR and 1771 non-DR controls. The findings demonstrated overall sensitivity (0.82, 95% CI: 0.78 ~ 0.85), specificity (0.84, 95% CI: 0.81 ~ 0.86), PLR (5.0, 95% CI: 4.2 ~ 5.9), NLR (0.22, 95% CI: 0.18 ~ 0.26), and the area under the summary ROC curve (0.90, 95% CI: 0.87 ~ 0.92). Furthermore, we performed subgroup analysis and found that panels of multiple miRNAs could enhance the pooled sensitivity (sensitivity, specificity, and AUC values were 0.89, 0.87, and 0.94, respectively).

**Conclusion:**

The meta-analysis showed that miRNAs can be used as potential diagnostic markers for DR, with high accuracy of diagnoses observed with the detection of miRNAs in plasma and serum.

## Introduction

Diabetic retinopathy (DR) is a diabetic microvascular complication (1) and an important cause of visual impairment and blindness in adults worldwide (2). Among individuals with diabetes, the global prevalence of DR is 22.27%. In 2020, the number of adults diagnosed with DR was estimated to be 103.12 million (3), and this number is expected to increase to 160.5 million by 2045 (4). The early stages of DR have no conscious symptoms, and as the extent of the disease progresses vision loss can occur to varying degrees, and if untreated progresses to the proliferative stage, irreversible vision loss can result, therefore, it is critical to prevent progression from the early stages of DR (5, 6). Fundus fluorescein angiography is the gold standard for the diagnosis of DR (7, 8), and fundoscopy, fundus photography, and optical coherence tomography have emerged in the clinical setting (8). However, because fluorescein angiography is invasive, time-consuming, and risky, and in addition, interpretation of fundoscopic examinations and photographic reports requires physicians with expertise and skills in diabetes-related eye diseases (9), these methods are not applicable to the routine screening of DR in clinical practice. In recent years, miRNAs have been shown to play an important role in various diabetic complications (10, 11). The application of circulating miRNAs as biomarkers of DR has garnered significant interest (12, 13). Therefore, the construction of circulating markers for early diagnosis of DR and timely treatment are fundamental goals to improve its diagnosis and treatment.

MicroRNAs (miRNAs) are a group of endogenous non-coding single-stranded small molecule RNAs of 19-24 nucleotides in length (14), which are involved in the post-transcriptional regulation of gene expression and are closely associated with various pathophysiological processes in the human body (15–17). Since abnormal alterations in miRNA expression precedes the expression of the corresponding regulated proteins, the abnormal expression of miRNAs may already be present when patients with diabetes have not yet developed retinal microvascular damage, or when the damage has not yet affected visual function (18). miRNAs are not only widely present in a variety of human tissues and cells, but can also bind to Argonaute proteins (19), which, in turn, prevents the degradation of RNA enzymes, allowing miRNAs to be stably expressed in serum, plasma, and other body fluids (20). This stability, ubiquity, high specificity, high sensitivity, and recent advances in the detection and analysis methods render miRNAs potentially useful novel biomarkers for DR diagnosis (21).

However, there is little consistency in previous studies; hence, no reliable conclusions can be drawn regarding the relationship between miRNAs and DR. In addition, most of the available data are derived from retrospective studies with a small sample size lacking quantitative criteria. Therefore, we performed a diagnostic meta-analysis for the first time to determine the potential diagnostic value of miRNAs in patients with DR.

## Materials and Methods

We have registered our protocol on PROSPERO (CRD42022323238) and can be found at: https://www.crd.york.ac.uk/prospero/. This meta-analysis follows the PRISMA statement of preferred reporting items for systematic evaluation and meta-analysis (**Supplementary Table 1**).

### Search Strategy

We searched PubMed, Cochrane Library, Embase, Web of Science, China Wanfang database, and China Knowledge Network (CNKI) database to identify relevant articles published from the time of database creation to April 10, 2022, without restricting the language of publication. A search strategy for diagnostic DR of circulating microRNAs was developed based on PICOS principles. We obtained all the medical subject headings (MeSH) and entry words on the National Center for Biotechnology Information (NCBI) website, and search terms included “Diabetic Retinopathy” OR “Diabetic Retinopathies” OR “Retinopathies, Diabetic” OR “Retinopathy, Diabetic” AND “MicroRNAs” OR “MicroRNA” OR “miRNAs” OR “Micro RNA” OR “RNA, Micro” OR “Primary MicroRNA” OR “MicroRNA, Primary” OR “Primary miRNA” OR “pri-miRNA” OR “pri miRNA” OR “RNA, Small Temporal” OR “Temporal RNA, Small” OR “stRNA” OR “Small Temporal RNA” OR “pre-miRNA” OR “pre miRNA” AND “sensitiv*” OR “sensitivity and specificity” OR “predictive” OR “value*” OR “accuracy*”. We searched PubMed using the following strategy:((((Diabetic Retinopathy[Title/Abstract]) OR (Diabetic Retinopathies[Title/Abstract])) OR (Retinopathies, Diabetic[Title/Abstract])) AND ((((((MicroRNAs[Title/Abstract]) OR (miRNA[Title/Abstract])) OR (Micro RNA[Title/Abstract])) OR (pri-miRNA[Title/Abstract])) OR (stRNA[Title/Abstract])) OR (miR[Title/Abstract]))) AND (sensitiv*[Title/Abstract] OR sensitivity and specificity[MeSH Terms] OR (predictive[Title/Abstract] AND value*[Title/Abstract]) OR predictive value of tests[MeSH Term] OR accuracy*[Title/Abstract]).

### Eligibility Criteria and Quality Assessment

Subjects were recruited with the following inclusion criteria: (1) all patients in the case group were diagnosed following clinically recognized diagnostic criteria; (2) the intervention was characterized by the diagnosis of DR performed using miRNA examination; (3) false positive (FP), true positive (TP), false negative (FN), and true negative (TN) could be derived directly or calculated from the literature. The exclusion criteria were set as follows: (1) non-human trials; (2) non-case-control studies; (3) reviews, letters, or conference proceedings; (4) insufficient data.

Two investigators used the Quality Assessment of Diagnostic Accuracy Studies-2 (QUADAS-2) (22) tool to independently assess the risk of bias and clinical applicability of included studies. The tool comprised four main components: selection of cases, trials to be evaluated, gold standard, and case flow and progression. Disagreements were resolved by agreement between the two investigators through negotiation or by involving a third reviewer.

### Data Extraction

Data and information were extracted independently by two investigators from eligible studies, including first author, year of publication, country, miRNAs, number of samples, internal reference, cut-off values, control source, sample source, assay method, miRNAs expression, AUC with 95% confidence intervals (CIs), sensitivity, specificity, TP, FP, FN, and TN.

### Statistical Analysis

We extracted TP, FP, FN, and TN values to calculate pooled sensitivity, specificity, positive likelihood ratio (PLR), negative likelihood ratio (NLR), diagnostic ratio (DOR), and corresponding 95% confidence interval (CI). Summary receiver operating characteristic (SROC) curves were plotted to calculate the area under the curve (AUC) to test the pooled diagnostic value of miRNAs. We used the chi-square test and I^2^ test to assess the heterogeneity between studies. Heterogeneity between studies was assessed using Cochran-Q test and I^2^ statistic for quantitative analysis, with *P*-value was less than 0.05 for Cochran-Q test and I^2^>50%, indicating significant heterogeneity between studies and selection of random-effect model (23, 24). We performed subgroup analysis and regression analysis to determine the source of heterogeneity. Sensitivity analysis was performed to determine the robustness of the meta-analysis results. Deeks’ funnel plots were used to examine publication bias. In addition, Fagan′s nomogram was drawn to further assess the diagnostic efficacy of miRNAs. Review Manager 5.3 was used to evaluate the quality of the literature, and Stata 14.0 was used to analyze all data. A *P*-value < 0.05 was considered statistically significant.

## Results

### Literature Search and Study Characteristics

**Figure 1:**
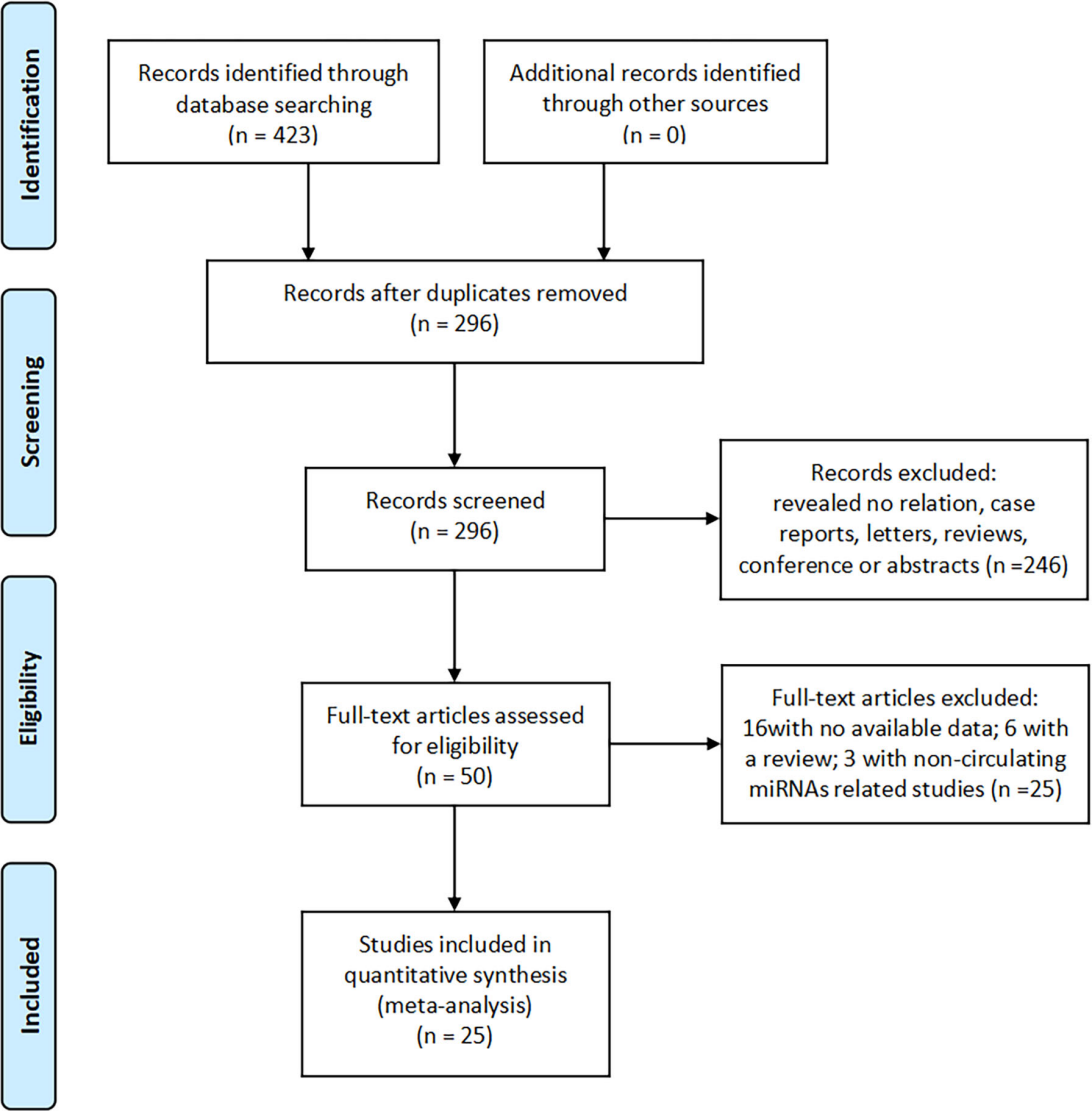
Flow diagram showing selection of studies for meta-analysis.

We searched 423 records in PubMed, Cochrane Library, Embase, Web of Science, China Wanfang database, and CNKI database. Of these, 296 duplicate studies were excluded. Subsequently, based on the titles and abstracts, 246 irrelevant studies, reviews, conference proceedings, and commentaries were excluded, and we evaluated 50 full-text articles and excluded 25 studies based on the exclusion criteria, including 16 studies with insufficient data and six reviews and three studies that were not related to miRNAs. The remaining 25 documents were included in the meta-analysis. The screening flow chart is shown in **Figure 1**.

In total, 25 documents (spanning years 2014 to 2022) included in this meta-analysis involved 52 studies (11, 18, 20, 25–46) (**Table 1**), all of which were case-control studies that included a total of 1987 patients with DR and 1771 non-DR controls, with the source of controls being diabetic patients not diagnosed with DR, healthy population, and patients with cataract. Among these 52 studies, 41 studies reported a single miRNA, and 11 studies discussed a panel of miRNAs. A total of 38 studies detected miRNAs in serum, 10 studies extracted miRNAs in plasma, and four studies extracted miRNAs from the aqueous humor. A total of four studies were conducted in Africa, two studies were conducted in Europe, and the remaining studies were conducted in Asian populations. In addition, 7 of the included studies involved the examination of miRNA-21, and miRNA-20b, miRNA-93, and miRNA-15 were examined in five studies.

**Table 1:** Characteristics of the included studies.

First author/Year	Ethnicity	miRNAs	Expression	Reference	Cut-off	Case/Control	Controls	Method	Specimen	Sen (%)	Spe (%)	AUC (95%CI)
Qing 2014 (23)	Asian	miR-21ˎ1179 miR-181c	up	U6	NA	30/30	HI	qRT-PCR	Serum	82	95	0.89 (0.86-0.96)
Fu 2017 (24)	Asian	miR-93	up	U6	1.42	98/80	DM	qRT-PCR	plasma	80.5	89.6	0.892 (0.824-0.961)
Fu 2017 (24)	Asian	miR-21	Up	U6	1.56	98/80	DM	qRT-PCR	plasma	70.2	90.8	0.836 (0.770-0.905)
Fu 2017 (24)	Asian	miR-93ˎmiR-21	Up	U6	NA	98/80	DM	qRT-PCR	plasma	86.4	91.3	0.937 (0.868-0.992)
Jiang 2017 (26)	Asian	miR-21	Up	U6	NA	124/180	DM/HI	qRT-PCR	plasma	66.1	90.4	0.825 (0.778-0.872)
Qin 2017 (25)	Asian	miR-126	down	U6 snRNA	8.43	81/59	HI	qRT-PCR	Serum	84.8	93.6	0.976
Zou 2017 (27)	Asian	miR-93	Up	U6	1.31	75/127	HI	qRT-PCR	plasma	73.3	89.2	0.866
Sun 2018 (28)	Asian	miR-21	Up	U6	NA	30/70	HI	qRT-PCR	plasma	81.8	86.7	0.797 (0.709-0.886)
Zheng 2018 (29)	Asian	miR-126	down	U6	≤0.64	110/80	HI	qRT-PCR	plasma	83.6	82.5	0.861
Liang 2018 (30)	Asian	has-miR-425-5p 7a5pˎmiR-28-3p has-miR-425-5p miR-novel-chr5_15976	up		NA	29/50	HI	qRT-PCR	Serum	92.7	87.5	0.937
Dai 2019 (31)	Asian	miR-451	Up	U6	1.86	60/60	DM	qRT-PCR	Serum	61	82.5	0.722
Dai 2019 (31)	Asian	miR-221	Up	U6	0.823	60/60	DM	qRT-PCR	Serum	71.2	85	0.823
Dai 2019 (31)	Asian	miR-200b	Up	U6	0.761	60/60	DM	qRT-PCR	Serum	81.4	72.3	0.761
Dai 2019 (31)	Asian	miR-451ˎmiR-221 miR-200b	Up	U6	NA	60/60	DM	qRT-PCR	Serum	93.2	77.5	0.938
Ma 2019 (32)	Asian	miR-93	up	U6	NA	76/45	HI	qRT-PCR	Serum	81	89	0.883 (0.813-0.950)
Ma 2019 (32)	Asian	miR-21	up	U6	NA	76/45	HI	qRT-PCR	Serum	71	90	0.845 (0.769-0.914)
Ma 2019 (32)	Asian	miR-93ˎmiR-21	Up	U6	NA	76/45	HI	qRT-PCR	Serum	87	92	0.946 (0.857-0.993)
Yu 2019 (17)	Asian	miR-19b	up	NA	NA	39/30	DM	qRT-PCR	Serum	87	80	0.78 (0.66-0.90)
Yu 2019 (17)	Asian	miR-221	up	NA	NA	39/30	DM	qRT-PCR	Serum	100	73	0.89 (0.81-0.97)
Yu 2019 (17)	Asian	miR-18b	up	NA	NA	39/30	DM	qRT-PCR	Serum	69	97	0.78 (0.67-0.90)
Li 2019 (33)	Asian	miR-4448ˎmiR-338-3p	down	NA	−43.869	10/11	T2DM	qRT-PCR	Serum	90	90	0.909
		miR-190a-5p										
		miR-485-5pˎmiR-9-5p										
Shaker 2019 (19)	African	miR-20b	down	SNORD 68	4.375	50/30	T2DM	qRT-PCR	Serum	62	60	0.858 (0.753-0.963)
Shaker2019 (19)	African	miR-17-3p	down	SNORD 68	0.2	50/30	T2DM	qRT-PCR	Serum	92	56.7	0.678 (0.535-0.821)
Ji 2019 (34)	Asian	miR-2116-5p	up	cel-miR-39	NA	45/45	T2DM	qRT-PCR	Serum	62.2	77.8	0.756
Ji 2019 (34)	Asian	miR-3197	up	cel-miR-39	NA	45/45	T2DM	qRT-PCR	Serum	93.3	91.1	0.966
Ji 2019 (34)	Asian	miR-2116-5p miR-3197	up	cel-miR-39	NA	45/45	T2DM	qRT-PCR	Serum	97.8	88.9	0.972
Lin 2020 (35)	Asian	miR-15	down	U6	0.63	105/50	HI	qRT-PCR	Serum	84.9	65.3	0.796 (0.724-0.857)
Lin 2020 (35)	Asian	miR-29a	up	U6	0.11	105/50	HI	qRT-PCR	Serum	53.8	79.6	0.677 (0.597-0.749)
Liu 2020 (36)	Asian	miR-15a	up	U6	219.20 copies/μL	34/40	CP	qRT-PCR	AH	82.4	61.5	0.762 (0.654-0.871)
Liu 2020 (36)	Asian	miR-16	up	U6	148.06 copies/μL	34/40	CP	qRT-PCR	AH	67.6	66.7	0.671 (0.546-0.797)
Liu 2020 (36)	Asian	miR-20b	up	U6	244.31 copies/μL	34/40	CP	qRT-PCR	AH	88.2	69.2	0.862 (0.780-0.943)
Liu 2020 (36)	Asian	miR-15aˎmiR-16 miR-20b	up	U6	NA	34/40	CP	qRT-PCR	AH	91.2	76.9	0.912 (0.848-0.976)
Liu 2020 (36)	Asian	miR-15a	up	U6	2.77	34/40	CP	qRT-PCR	Serum	76.5	76.9	0.798 (0.695-0.901)
Liu 2020 (36)	Asian	miR-16	up	U6	2.39	34/40	CP	qRT-PCR	Serum	70.6	64.1	0.688 (0.565-0.810)
Liu 2020 (36)	Asian	miR-20b	up	U6	3.42	34/40	CP	qRT-PCR	Serum	79.4	82.1	0.886 (0.806-0.967)
Liu 2020 (36)	Asian	miR-15a miR-16ˎmiR-20b	up	U6	NA	34/40	CP	qRT-PCR	Serum	79.4	92.3	0.931 (0.872-0.990)
Yin 2020 (37)	Asian	miR-210	up	U6	1.905	110/60	HI	qRT-PCR	Serum	95.5	95	0.991
Yin 2020 (37)	Asian	miR-210	up	U6	2.335	110/40	DM	qRT-PCR	Serum	83.6	80	0.892
Wan 2020 (38)	Asian	miR-409-5p	up	U6	NA	115/102	T2DM	qRT-PCR	Serum	83.3	57.4	0.757 (0.693-0.820)
Wan 2020 (38)	Asian	miR-216a	down	U6	NA	115/102	T2DM	qRT-PCR	Serum	47.1	87.8	0.703 (0.633-0.772)
Wan 2020 (38)	Asian	miR-409-5p	up	U6	NA	115/100	HI	qRT-PCR	Serum	90	79.1	0.892 (0.848-0.935)
Wan 2020 (38)	Asian	miR-216a	down	U6	NA	115/100	HI	qRT-PCR	Serum	71	87.8	0.859 (0.810-0.908)
Hu 2021 (39)	Asian	miR-29c	up	U6	1.31	65/64	HI	qRT-PCR	Serum	64.6	78.7	0.716 (0.638-0.785)
Liu 2021 (40)	Asian	miR-211	up	U6	2.23	90/90	DM/HI	qRT-PCR	plasma	72.4	75	0.839 (0.785-0.894)
Sun 2021 (41)	Asian	miR-320	down	NA	NA	179/83	DM	qRT-PCR	Serum	63.1	91.6	0.788 (0.734-0.842)
Santovito 2021 (42)	European	miR-25-3p miR-320b miR-495-3p	up/down	miR-19-5p miR-125a-5p	NA	20/20	T2DM/HI	qRT-PCR	plasma	85	85	0.931 (0.853-1.000)
Santovito 2021 (42)	European	miR-320b miR-495-3p	up/down	miR-19-5p miR-125a-5p	NA	20/20	T2DM/HI	qRT-PCR	plasma	83	79	0.847 (0.722-0.972)
Wang 2021 (43)	Asian	miR-374a	up	snoRNA U6	1.659	137/70	T2DM	qRT-PCR	Serum	82.9	80.3	0.892
Liu 2022 (44)	Asian	miR-425-5p	up	U6	1.565	100/60	HI	qRT-PCR	Serum	91.7	84	0.907
Liu 2022 (44)	Asian	miR-425-5p	up	U6	1.71	100/35	T2DM	qRT-PCR	Serum	85.7	78	0.833
Salem 2022 (14)	African	miR-181C	up	U6	NA	60/60	HI	qRT-PCR	Serum	90	100	0.983 (0.94-1.0)
Salem 2022 (14)	African	miR-1179	up	U6	NA	60/60	HI	qRT-PCR	Serum	90	80	0.927 (0.82-1.0)

up, upregulated; down, downregulated; NA, not available; HI, health individuals; CP, cataract patients; AH, aqueous humor; Sen, sensitivity; Spe, specificity; AUC, area under the curve; CI, confidence interval.

### Quality Assessment

**Figure 2:**
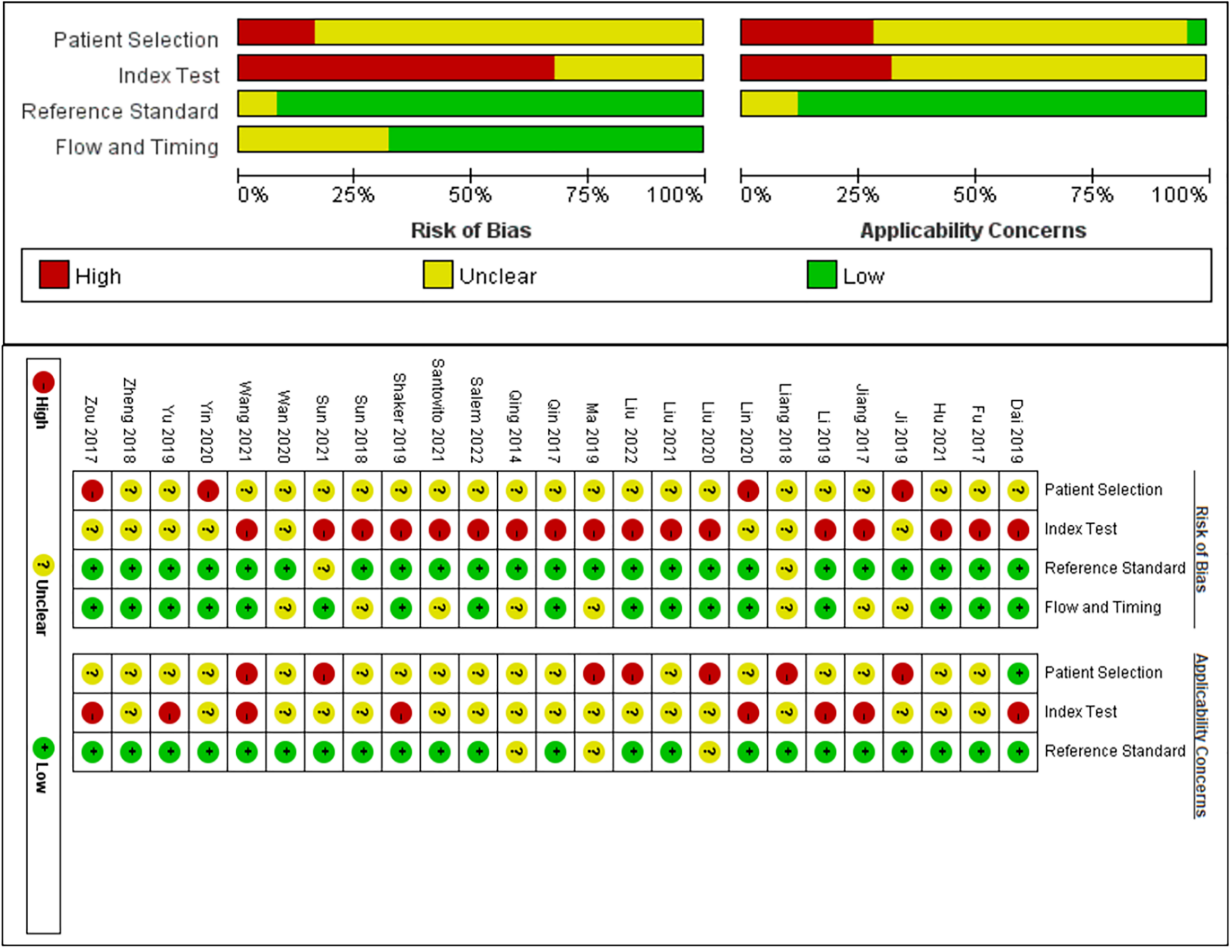
Quality Assessment of Diagnostic Accuracy Studies (QUADAS)-2 assessment for risk of bias and applicability. Red, yellow and green indicate high, unclear and low risk respectively.

The quality of the included studies was evaluated using the QUADAS-2, and the results were analyzed by applying Review Manager 5.3. The results are shown in **Figure 2**. Most of the studies included in our meta-analysis were continuous and time scaled. Given that all patients with DR included in the study were diagnosed by clinically recognized diagnostic criteria, all trials involved case-control studies, introducing high or unclear risks in the selection field.

### Diagnostic Accuracy of MiRNAs in DR

**Figure 3:**
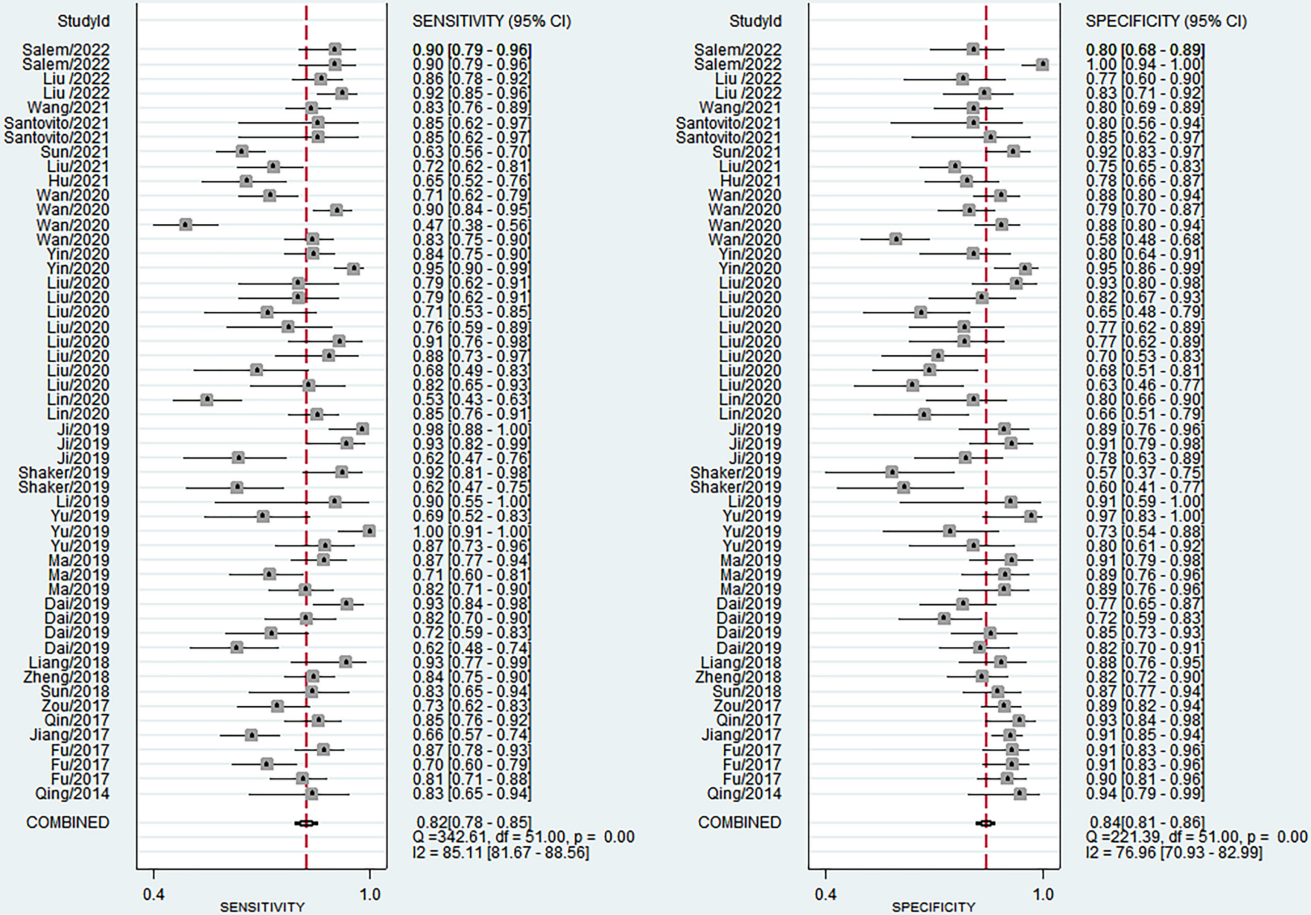
Forest plots of studies examining microRNAs used in the diagnosis of diabetic retinopathy.

**Figure 4:**
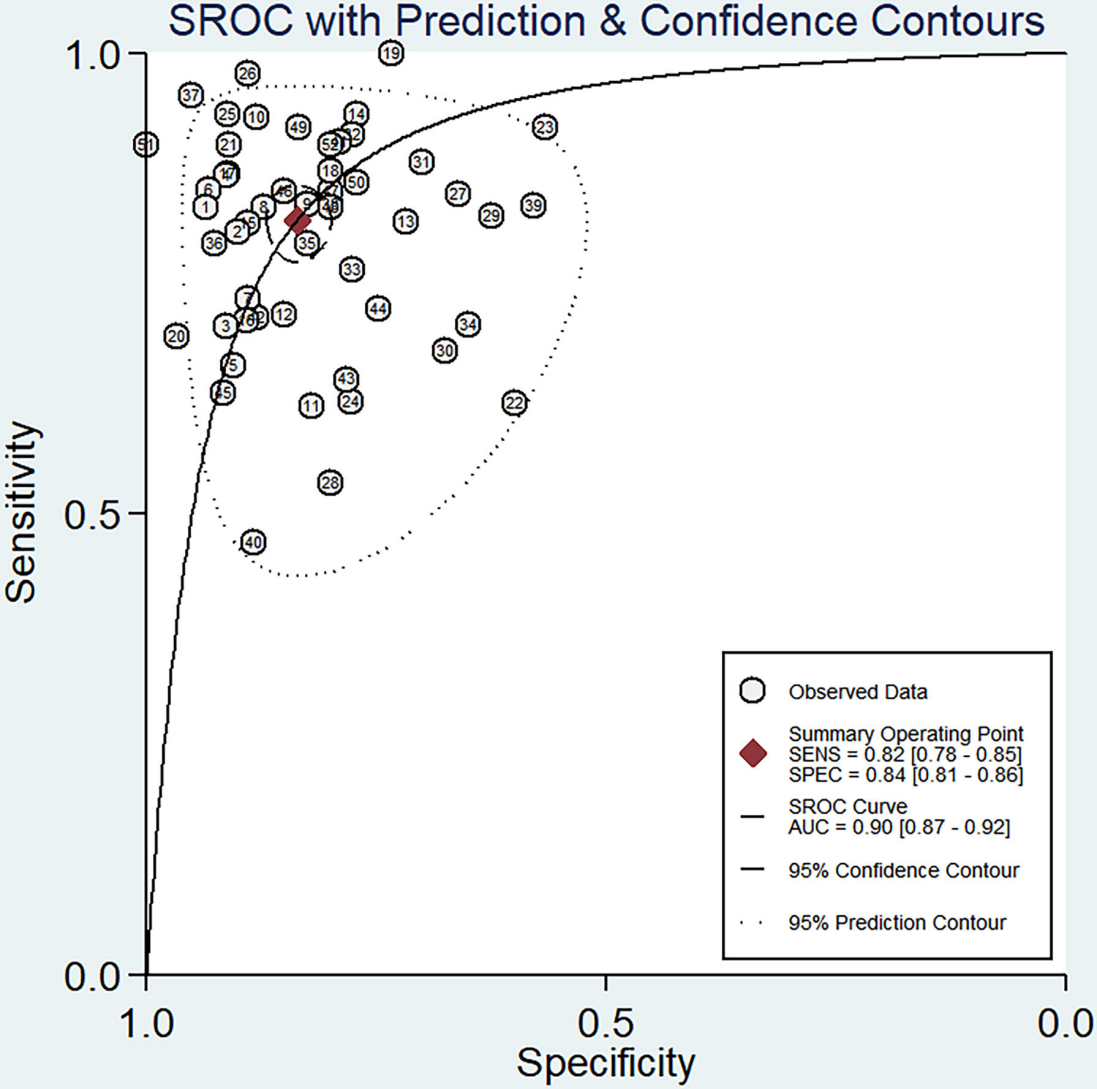
Summary receiver operator characteristic (SROC) curve examining the overall accuracy of miRNAs in the diagnosis of diabetic retinopathy.

**Figure 5:**
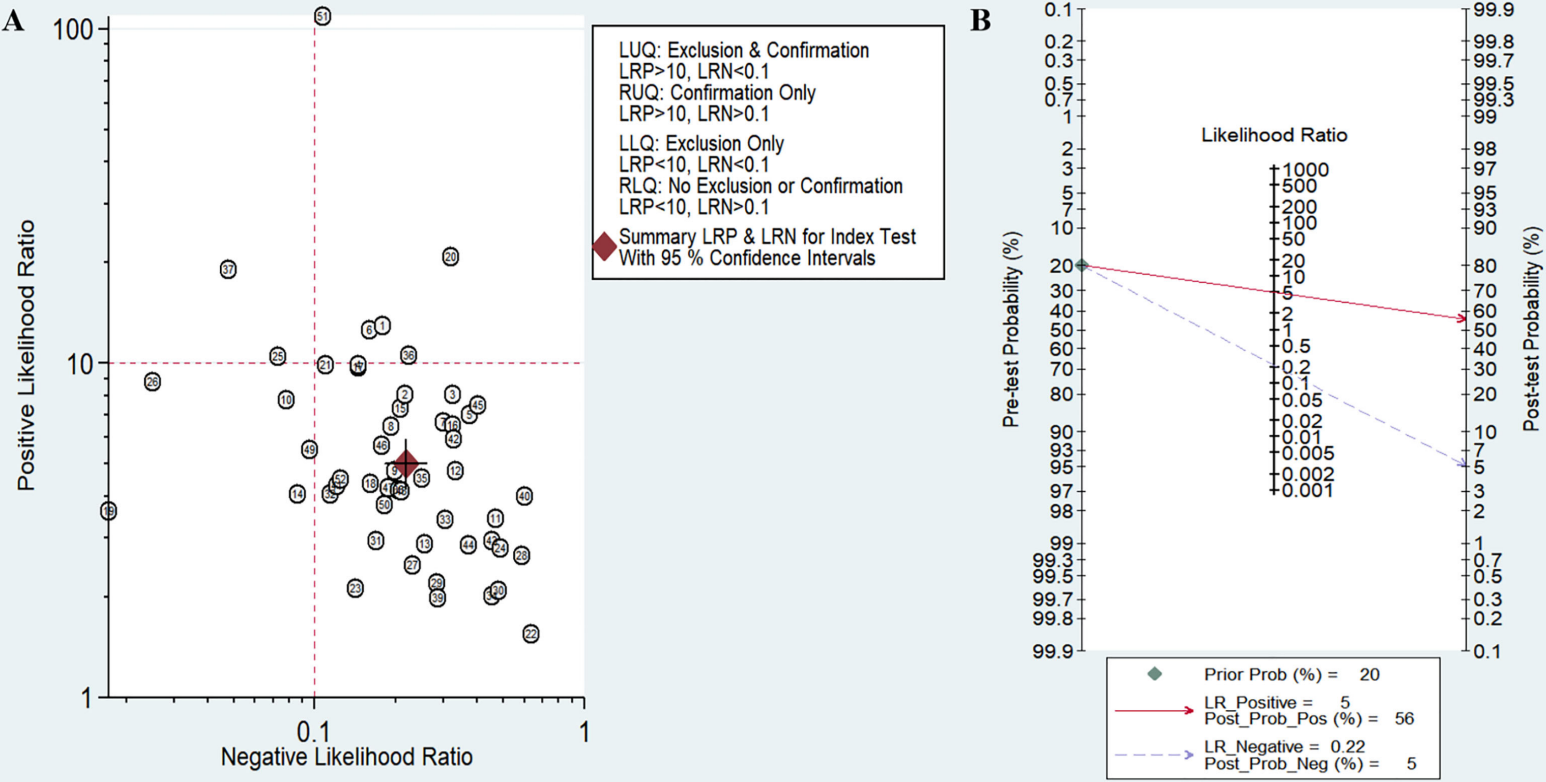
Assessment of clinical applicability of miRNAs for diagnosis of diabetic retinopathy (DR). **(A)** Summary of positive likelihood ratio and negative likelihood ratio for diagnosis of DR; **(B)** Fagan’s nomogram of miRNA studies for diagnosis of DR.

The sensitivity and specificity of miRNAs in diagnosing DR are shown in **Figure 3**. Forest plots of pooled data showed I^2^ = 85.11% for sensitivity and I^2^ = 76.96% for specificity, suggesting a large heterogeneity between studies, and a random effects model was used to estimate the diagnostic performance of miRNAs in DR. The results for diagnostic accuracy of miRNA for DR were as follows: sensitivity, 0.82 (95% CI: 0.78-0.85); specificity, 0.84 (95% CI: 0.81-0.86); NLR, 0.22 (95% CI: 0.18 -0.26); PLR, 5.0 (95% CI: 4.2-5.9), and DOR, 23 (95% CI: 17-31). In addition, we plotted the SROC curve to assess the diagnostic accuracy (**Figure 4**). The AUC was 0.90 (95% CI: 0.87-0.92), which indicated a strong overall accuracy of miRNAs in the diagnosis of DR. Aiding in the process of clinical decision during diagnosis is an important value of biomarkers. Therefore, we plotted a graph (**Figure 5**) based on the combination of PLR and NLR to determine their clinical applicability. PLR >10 and NLR <0.1 represent high diagnostic accuracy. We observed superior diagnostic efficacy of miRNAs in two studies in the articles by Yin et al. and Ji et al. (25, 37). Therefore, miR-3197 and miR-210 may be promising miRNAs and should be further examined in future studies (**Figure 5A**). MiRNAs with high diagnostic accuracy were also confirmed by Fagan′s nomogram, with post-test probabilities of 0.56 and 0.5 for PLR and NLR, respectively, when the pre-test probability was set at 20% (**Figure 5B**).

### Subgroup Analyses, Meta-Regression, and Sensitivity Analyses

To explore the potential heterogeneity, we performed subgroup analysis (**Table 2**) and regression analysis, grouped according to miRNA profiling, sample size, sample source, miRNA expression, ethnicity, internal reference and cut-off values setting. Notably, the diagnostic accuracy of miRNA panel was higher compared to single miRNA. The sensitivity, specificity, PLR, NLR, DOR and AUC values for single miRNAs and miRNA panel were 0.80 (95% CI: 0.76-0.83), 0.83 (95% CI: 0.79-0.86), 4.6 (95% CI: 3.8-5.5), 0.24 (95% CI: 0.20-0.30), 19 (95% CI: 14-26) and 0.88 (95% CI: 0.85-0.91) and 0.89 (95% CI: 0.85-0.92), 0.87 (95% CI: 0.83-0.91), 7.1 (95% CI: 5.2-9.6), 0.13 (95% CI: 0.09-0.17), 56 (95% CI: 37-86) and 0.94 (95% CI: 0.92-0.96), respectively. The results of the diagnostic accuracy of DR for sample size <100 and sample ≥100 are listed as follows: the sensitivity was 0.82 (95% CI: 0.79-0.85) vs. 0.80 (95% CI: 0.72-0.86), specificity was 0.84 (95% CI: 0.80-0.87) vs. 0.83 (95% CI: 0.78-0.87), PLR was 5.1 (95% CI: 4.1-6.3) vs. 4.7 (95% CI: 3.6-6.2), NLR was 0.21 (95% CI: 0.17-0.26) vs. 0.24 (95% CI: 0.17-0.34), DOR was 24 (95% CI: 17-35) vs. 20 (95% CI: 12-32), and AUC was 0.90 (95% CI: 0.87-0.92) vs. 0.89 (95% CI: 0.85-0.91). No significant difference was observed in the overall diagnostic accuracy of DR for sample size <100 compared to that observed with sample size ≥100. In plasma samples, the values corresponding to sensitivity, specificity, PLR, NLR, DOR, and AUC were 0.78 (95% CI: 0.73-0.82), 0.87 (95% CI: 0.83-0.90), 6.1 (95% CI: 4.7-7.9), 0.25 (95% CI: 0.20-0.31), 24 (95% CI: 16-36), and 0.90 (95% CI: 0.87-0.92), respectively. In serum samples, the values corresponding to sensitivity, specificity, PLR, NLR, DOR, and AUC were 0.83 (95% CI: 0.78-0.86), 0.84 (95% CI: 0.80-0.87), 5.1 (95% CI: 4.1-6.3), 0.21 (95% CI: 0.16-0.26), 25 (95% CI: 17-36), and 0.90 (95% CI: 0.87-0.92), respectively. In aqueous humor, the values corresponding to sensitivity, specificity, PLR, NLR, DOR, and AUC were 0.83 (95% CI: 0.72-0.91), 0.69 (95% CI: 0.61-0.87), 2.7 (95% CI: 2.0-3.7), 0.24 (95% CI: 0.13-0.43), 11 (95% CI: 5-26), and 0.77 (95% CI: 0.73-0.80), respectively. Interestingly, miRNA expression levels were also correlated with diagnostic value, and our results showed that miRNAs with increased expression exhibited better diagnostic value. In the group demonstrating increased expression of miRNAs, the corresponding values for sensitivity, specificity, PLR, NLR, DOR and AUC were 0.83 (95% CI: 0.79-0.86), 0.84 (95% CI: 0.81-0.87), 5.1 (95% CI: 4.2-6.2), 0.21 (95% CI: 0.17-0.25), 25 (95% CI: 18-35) and 0.90 (95% CI: 0.87-0.92), respectively. In addition, ethnicity had an impact on the diagnostic value of miRNAs, compared to the Asian population, the non-Asian populations had higher diagnostic accuracy, for sensitivity 0.86 (95% CI: 0.76-0.92), specificity 0.83 (95% CI: 0.61-0.94), PLR 5.0 (95% CI: 1.9 -13.1), NLR 0.17 (95% CI: 0.09-0.32), DOR 29 (95% CI: 7-124), and AUC 0.91 (95% CI: 0.88-0.93). Surprisingly, most studies selected U6 as an internal reference, yet the diagnostic value of the selected other miRNA internal reference groups was more significant than the U6 group, with sensitivity, specificity, PLR, NLR, DOR and AUC for the U6 group, respectively, of 0.80 (95% CI: 0.77-0.83), 0.84 (95% CI: 0.81-0.87), 5.0 (95%CI: 4.1-6.0), 0.24 (95%CI: 0.20-0.28), 21 (95%CI: 16-29) and 0.89 (95%CI: 0.86-0.92), respectively, for the non-U6 group sensitivity, specificity, PLR, NLR, DOR and AUC, respectively, were 0.88 (95% CI: 0.76-0.94), 0.81 (95% CI: 0.71-0.88), 4.5 (95% CI: 2.8-7.3), 0.15 (95% CI: 0.07 -0.33), 29 (95% CI: 9-91) and 0.90 (95% CI: 0.87-0.92), while studies without an explicitly given internal reference had higher diagnostic value, with sensitivity, specificity, PLR, NLR, DOR and AUC were 0.90 (95% CI: 0.70-0.97), 0.86 (95% CI: 0.72-0.94), 6.4 (95% CI: 3.3-12.6), 0.12 (95% CI: 0.04-0.36), 56 (95% CI: 19-164) and 0.93 (95% CI: 0.91-0.95). In addition, studies with optimal cut-off values had pooled results for sensitivity, specificity, PLR, NLR, DOR and AUC of 0.80 (95% CI: 0.75-0.83), 0.80 (95% CI: 0.76-0.84), 4.0 (95% CI: 3.2-4.9), 0.26 (95% CI: 0.21-0.32), 15 (95% CI: 11-23) and 0.87 (95% CI: 0.83-0.89), while studies without cut-off values showed better diagnostic value with sensitivity of 0.84 (95%CI: 0.79–0.88), specificity of 0.87 (95%CI: 0.83–0.90), PLR of 6.4 (95%CI: 5.0–8.1), NLR of 0.18 (95%CI: 0.14–0.24), DOR of 35 (95%CI: 23–53), and AUC of 0.92 (95%CI: 0.89–0.94), respectively.

**Table 2:** Summary estimates of diagnostic power and their 95% confidence intervals.

Subgroup	No. studies	Sen (95% CI)	Spe (95% CI)	PLR (95% CI)	NLR (95% CI)	DOR (95% CI)	AUC (95% CI)
MiRNA proliling							
Single miRNA	41	0.80 (0.76-0.83)	0.83 (0.79-0.86)	4.6 (3.8-5.5)	0.24 (0.20-0.30)	19 (14-26)	0.88 (0.85-0.91)
Multiple miRNAs	11	0.89 (0.85-0.92)	0.87 (0.83-0.91)	7.1 (5.2-9.6)	0.13 (0.09-0.17)	56 (37-86)	0.94 (0.92-0.96)
Sample size							
<100	38	0.82 (0.79-0.85)	0.84 (0.80-0.87)	5.1 (4.1-6.3)	0.21 (0.17-0.26)	24 (17-35)	0.90 (0.87-0.92)
≥100	14	0.80 (0.72-0.86)	0.83 (0.78-0.87)	4.7 (3.6-6.2)	0.24 (0.17-0.34)	20 (12-32)	0.89 (0.85-0.91)
Specimen							
Plasma	10	0.78 (0.73-0.82)	0.87 (0.83-0.90)	6.1 (4.7-7.9)	0.25 (0.20-0.31)	24 (16-36)	0.90 (0.87-0.92)
Serum	38	0.83 (0.78-0.86)	0.84 (0.80-0.87)	5.1 (4.1-6.3)	0.21 (0.16-0.26)	25 (17-36)	0.90 (0.87-0.92)
aqueous humor	4	0.83 (0.72-0.91)	0.69 (0.61-0.87)	2.7 (2.0-3.7)	0.24 (0.13-0.43)	11 (5-26)	0.77 (0.73-0.80)
Regulation mode							
Up-regulate	41	0.83 (0.79-0.86)	0.84 (0.81-0.87)	5.1 (4.2-6.2)	0.21 (0.17-0.25)	25 (18-35)	0.90 (0.87-0.92)
Down-regulate	9	0.77 (0.66-0.85)	0.82 (0.73-0.89)	4.3 (2.8-6.6)	0.28 (0.19-0.42)	15 (8-29)	0.87 (0.83-0.89)
Ethnicity							
Asian	46	0.81 (0.78-0.85)	0.84 (0.81-0.86)	5.0 (4.3-5.9)	0.22 (0.18-0.27)	23 (17-30)	0.90 (0.87-0.92)
Non-Asian	6	0.86 (0.76-0.92)	0.83 (0.61-0.94)	5.0 (1.9-13.1)	0.17 (0.09-0.32)	29 (7-124)	0.91 (0.88-0.93)
Internal reference							
U6	40	0.80 (0.77-0.83)	0.84 (0.81-0.87)	5.0 (4.1-6.0)	0.24 (0.20-0.28)	21 (16-29)	0.89 (0.86-0.92)
Non-U6	8	0.88 (0.76-0.94)	0.81 (0.71-0.88)	4.5 (2.8-7.3)	0.15 (0.07-0.33)	29 (9-91)	0.90 (0.87-0.92)
NA	4	0.90 (0.70-0.97)	0.86 (0.72-0.94)	6.4 (3.3-12.6)	0.12 (0.04-0.36)	56 (19-164)	0.93 (0.91-0.95)
Cut-off values							
Given	26	0.80 (0.75-0.83)	0.80 (0.76-0.84)	4.0 (3.2-4.9)	0.26 (0.21-0.32)	15 (11-23)	0.87 (0.83-0.89)
NA	26	0.84 (0.79-0.88)	0.87 (0.83-0.90)	6.4 (5.0-8.1)	0.18 (0.14-0.24)	35 (23-53)	0.92 (0.89-0.94)

Sen, sensitivity; Spe, specificity; PLR, positive likelihood ratio; NLR, negative likelihood ratio; DOR, diagnostic odds ratio; AUC, area under the curve; CI, confidence interval; NA, not available.

**Figure 6:**
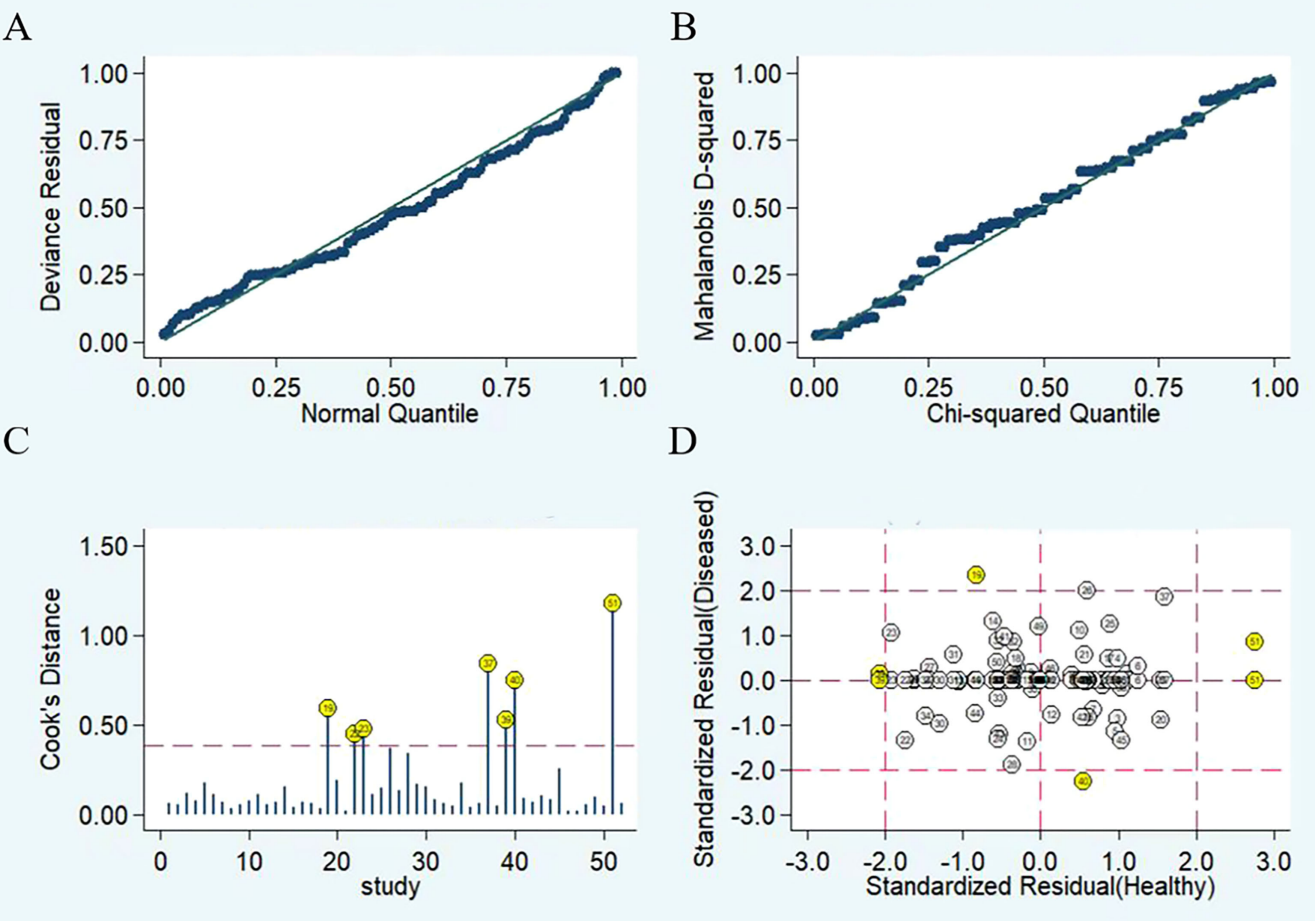
Diagram of sensitivity analysis showing **(A)** goodness-of-fit; **(B)** bivariate normality; **(C)** influence analysis; **(D)** outlier detection.

The results of the sensitivity analysis are shown in **Figure 6**. The goodness of fit (**Figure 6A**) and bivariate normality (**Figure 6B**) showed that the random effects model was suitable. Influence analysis identified that studies of Yu et al, Shaker et al, Yin et al, Wan et al, and Salem et al. were the most dominant studies in weight (**Figure 6C**). Outlier detection implied that heterogeneity may be attributed to the data corresponding to studies of Yu et al, Wan et al, and Salem et al. (**Figure 6D**). After excluding four outlier studies, the I^2^ value for heterogeneity decreased to 3.98% for sensitivity and 7.48% for specificity. There was no significant change in the pooled results for diagnostic efficacy (**Table 3**).

**Table 3:** Diagnostic performance of miRNAs in DR.

Analysis	Overall	Outliers excluded
No. of studies	52	48
Sen (95% CI)	0.82 (0.78-0.85)	0.81 (0.78-0.84)
Spe (95% CI)	0.84 (0.81-0.86)	0.83 (0.81-0.86)
PLR (95% CI)	5.0 (4.2-5.9)	4.9 (4.2-5.8)
NLR (95% CI)	0.22 (0.18-0.26)	0.22 (0.19-0.26)
DOR (95% CI)	23 (17-31)	22 (17-30)
AUC (95% CI)	0.90 (0.87-0.92)	0.89 (0.86-0.92)

Sen, sensitivity; Spe, specificity; PLR, positive likelihood ratio; NLR, negative likelihood ratio; DOR, diagnostic odds ratio; AUC, area under the curve; CI, confidence interval.

**Figure 7:**
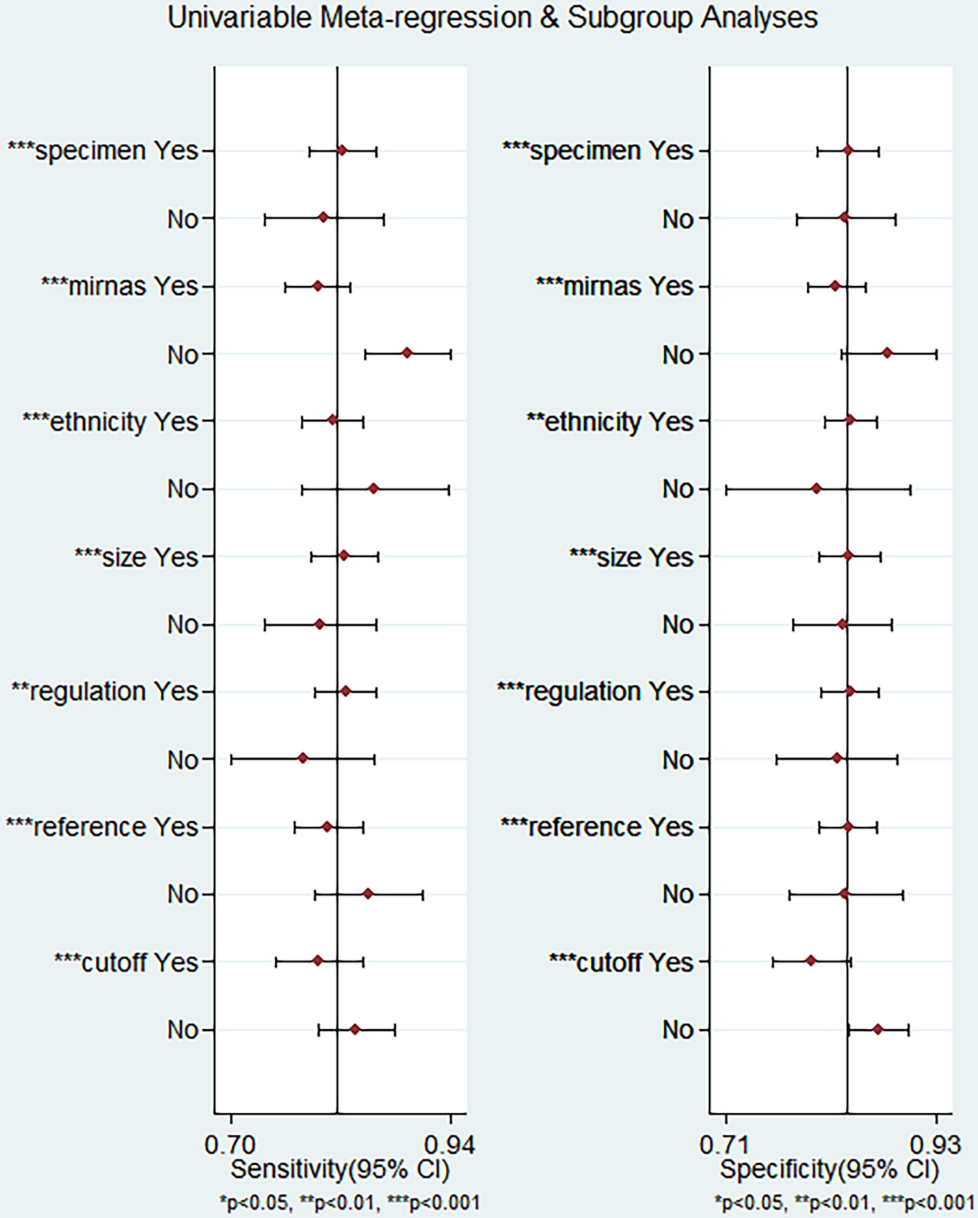
Meta-regression analysis for examining sensitivity and specificity of miRNAs for the diagnosis of diabetic retinopathy.

We performed meta-regression analysis with ethnicity, miRNA profiling, sample source, sample size, miRNA expression, internal reference and cut-off values as independent variables to explore the sources of heterogeneity. The results are shown in **Figure 7**. For sensitivity and specificity, all seven independent variables were statistically significant, indicating that ethnicity, miRNA profiling, sample source, sample size, miRNA expression, internal reference and cut-off values were sources of heterogeneity.

### Publication Bias

**Figure 8:**
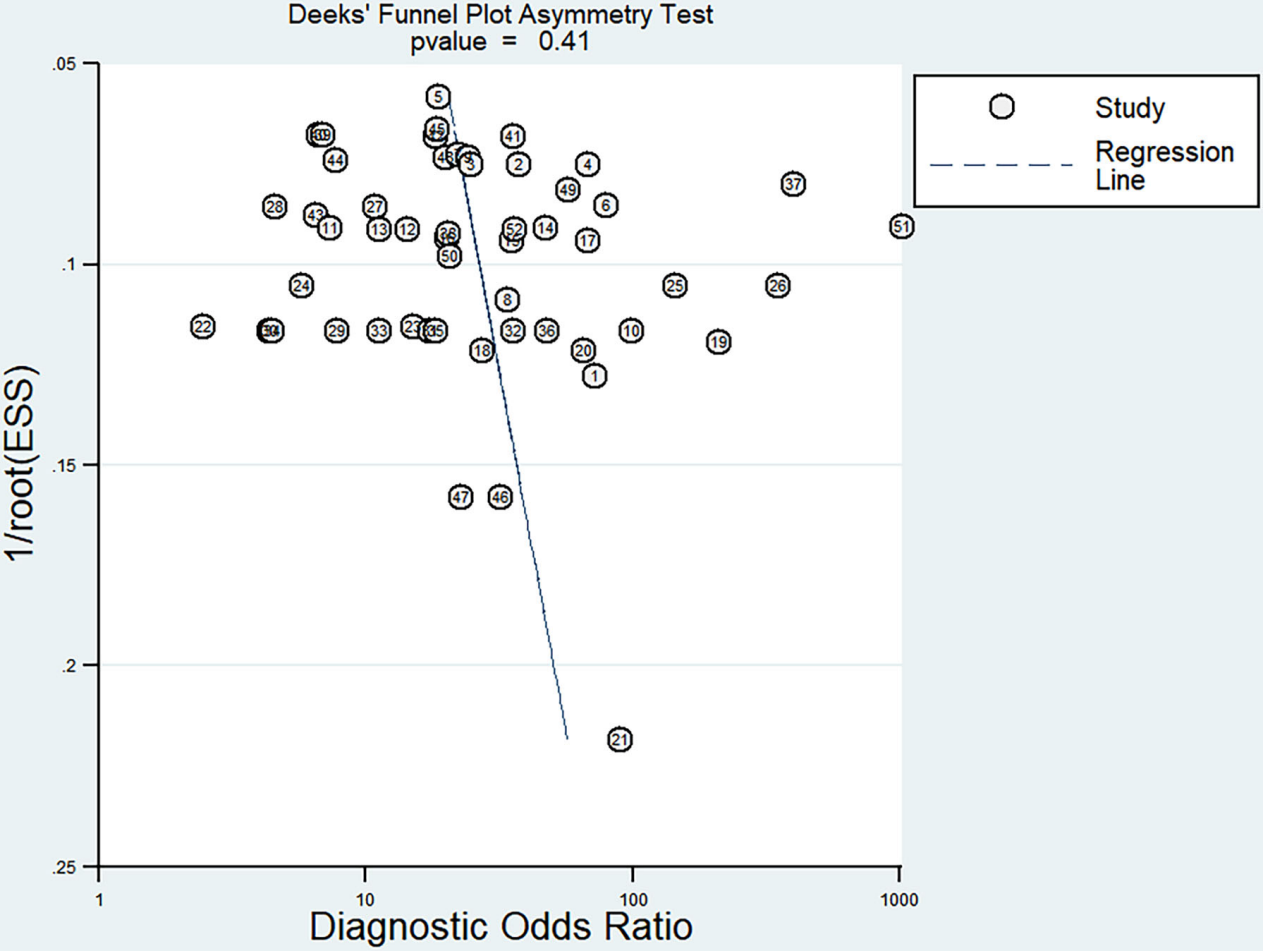
Funnel plot for determining publication bias.

Deek’s funnel plot was plotted for the 25 included papers (**Figure 8**). The studies were relatively symmetrically distributed on both sides of the regression line with *P*=0.41, and the differences were not statistically significant, indicating that there was no publication bias in the included data.

## Discussion

This meta-analysis of 25 articles including 1987 patients with DR and 1771 non-DR controls showed that miRNAs demonstrate high sensitivity (0.82, 95% CI: 0.78-0.85) and specificity (0.84, 95% CI: 0.81-0.86) in the diagnosis of DR. The pooled PLR of 5.0 indicates a 5.0-fold increase in the probability of an individual being diagnosed with DR. In addition, the NLR was 0.22, implying that the probability of subjects being diagnosed with DR was only 22%. DOR is an indicator of discriminatory test performance (47), and a DOR value greater than 1 indicates a better diagnostic test, with a DOR of 23, representing the ability of the miRNA to effectively discriminate between DR patients and non-DR control populations. However, due to heterogeneity among the included studies, we explored confounding factors by subgroup analysis and meta-regression analysis. Subgroup analysis revealed miRNA profiling, sample size, sample source, miRNA expression, ethnicity, internal reference and cut-off values setting as sources of heterogeneity.The combined detection of pooled miRNAs demonstrated better diagnostic accuracy than single miRNAs, with an AUC value of 0.94 for the miRNA group and 0.88 for single miRNAs. These results are consistent with previous findings by Zhou et al. on differential expression of miRNA in patients with DR (48), which may be explained by multiple gene mutations and epigenetic genetic abnormalities that are involved in the development of DR (49). Therefore, miRNA panel may be more suitable as a diagnostic biomarker for DR, which is the future development trend. One subgroup analysis that requires specific consideration showed that the sensitivity of miRNAs for diagnosis was higher in the non-Asian population (0.86) than in the Asian population (0.81). However, data from only six studies were available for the non-Asian group compared with 46 studies for the Asian group. Hence, we suggest that this difference may be attributed to the smaller number of studies performed with non-Asian populations. In addition, we found that the AUC values of miRNAs in serum, plasma, and aqueous humor were 0.90, 0.90, and 0.77, respectively, indicating that the detection of miRNAs in serum and plasma samples has high accuracy. Since the studies we included all used qRT-PCR to detect miRNAs levels, it was necessary to select appropriate internal reference genes for standardization, and we were surprised to find that although most studies used U6 as an internal reference gene, miRNAs showed higher diagnostic power with the analysis of other materials referred to as non-U6 references. qRT-PCR is the most widely used method for circulating miRNA expression profiling, and the accurate and reliable interpretation of its results depends heavily on the use of suitable reference genes for normalization, but the selection of suitable internal reference genes is still controversial (50), and the future selection of uniform and highly stable internal reference is essential to eliminate or minimize abiotic variation between test samples. Furthermore, in terms of clinical applicability, Fagan′s nomogram demonstrate promising results, with post-test probabilities of 0.56 and 0.5 for PLR and NLR, respectively, when the pre-test probability was set at 20%. This finding indicated that when the samples tested positive for the presence of miRNAs, patients had a 56% probability of developing DR, while the post-test probability of disease was reduced to 5% when the samples were tested negative for miRNAs. Among these studies, the expression of circulating miRNA-21 was reported to be upregulated in five articles. The sensitivity of miR-21 alone for the diagnosis of DR ranged from 0.661 to 0.818, and the specificity ranged from 0.867 to 0.908. Four studies analyzed miRNA-21 alone, and our meta-analysis of the diagnostic efficacy of miRNA-21 showed sensitivity, specificity, PLR, NLR, DOR, and AUC values of 0.70, 0.90, 6.9, 0.33, 21, and 0.89, respectively. Chen et al. demonstrated the protective effect of miR-21 silencing on neovascularization and inflammation in DR through a “knockdown” experiment (51). miR-21 may be an important regulator of reactive oxygen species homeostasis and antioxidant pathways, interfering with superoxide dismutase 2 expression and thereby affecting the antioxidant response system, which is one of the major causes of cellular damage (52, 53). Therefore, miRNA-21 is considered an early predictor of reactive oxygen species-mediated injury in individuals at high risk for diabetes and has the potential to be an excellent marker for the diagnosis of DR.

These are several strengths of this meta-analysis. This is the first meta-analysis to perform a detailed assessment of the diagnostic value of miRNAs in DR. A more comprehensive list of miRNAs was analyzed in this study. The study also delineated miRNA subtypes that require further examination. Secondly, subgroup analysis and meta-regression analysis were performed to explore the heterogeneity of the studies included based on the high degree of heterogeneity.

Some limitations of this meta-analysis should be highlighted. First, the cutoff values of miRNAs in the included studies differed, which could lead to heterogeneity. Second, we did not assess differences in the accuracy of miRNA detection at different stages of DR for the diagnosis of DR due to a lack of sufficient data. Third, most of the included studies were based on Asian populations, which may contribute to ethnic bias. Fourth, this meta-analysis only included articles published in English and Chinese, which may also contribute to unavoidable bias. Fifth, most studies are retrospective case-control studies, increasing the risk of bias in the quality assessment of patient selection domains, in addition to the fact that there is no consensus on the selection of uniform and stable internal reference genes, which may lead to inconsistent results in the relative quantitative analysis of miRNAs. In addition, the relatively small sample size of each study may have limited the statistical power. Regrettably, due to the lack of a large number of similar miRNAs to pool the results, it is not yet possible to identify specific single miRNA or miRNAs panel as the best diagnostic biomarkers for DR. Therefore, based on the above limitations, these findings need to be interpreted with caution, and the results of our meta-analysis need to be further confirmed by well-designed studies with larger sample sizes in the future.

In conclusion, the current evidence suggests that miRNAs have significant diagnostic value in predicting DR and may be employed as effective non-invasive biomarkers for DR. Furthermore, miRNA panels have higher diagnostic potency than individual miRNAs. However, quality studies with large samples sizes should be conducted to validate our results and confirm the clinical value of miRNAs for patients with DR.

## Data Availability Statement

The original contributions presented in the study are included in the article/**Supplementary Material**. Further inquiries can be directed to the corresponding author.

## Author Contributions

LM and YW wrote the manuscript, responsible for literature retrieval and statistical analysis, ZL participate in editing manuscripts, and QW participate in discussion and review manuscripts. All authors read and approved the final version of the manuscript.

## Funding

This project was supported by the major project of Chinese Medical Association Fund (No.TW-2018P003).

## Conflict of Interest

The authors declare that the research was conducted in the absence of any commercial or financial relationships that could be construed as a potential conflict of interest.

## Publisher’s Note

All claims expressed in this article are solely those of the authors and do not necessarily represent those of their affiliated organizations, or those of the publisher, the editors and the reviewers. Any product that may be evaluated in this article, or claim that may be made by its manufacturer, is not guaranteed or endorsed by the publisher.

## References

[1] Nebbioso M Lambiase A Armentano M Tucciarone G Sacchetti M Greco A. Diabetic Retinopathy, Oxidative Stress, and Sirtuins: An in Depth Look in Enzymatic Patterns and New Therapeutic Horizons. Surv Ophthalmol (2022) 67(1):168–83. doi: 10.1016/j.survophthal.2021.04.003 33864872

[2] Stitt AW Curtis TM Chen M Medina RJ McKay GJ Jenkins A. The Progress in Understanding and Treatment of Diabetic Retinopathy. Prog Retinal Eye Res (2016) 51:156–86. doi: 10.1016/j.preteyeres.2015.08.001 26297071

[3] Teo ZL Tham YC Yu M Chee ML Rim TH Cheung N. Global Prevalence of Diabetic Retinopathy and Projection of Burden Through 2045: Systematic Review and Meta-Analysis. Ophthalmology (2021) 128(11):1580–91. doi: 10.1016/j.ophtha.2021.04.027 33940045

[4] Pu S Xu Y Li X Yu Z Zhang Y Tong X. Lncrnas-Modulators of Neurovascular Units in Diabetic Retinopathy. Eur J Pharmacol (2022) 925:174937. doi: 10.1016/j.ejphar.2022.174937 35430212

[5] Lopez-Contreras AK Martinez-Ruiz MG Olvera-Montano C Robles-Rivera RR Arevalo-Simental DE Castellanos-Gonzalez JA. Importance of the Use of Oxidative Stress Biomarkers and Inflammatory Profile in Aqueous and Vitreous Humor in Diabetic Retinopathy. Antioxidants-Basel (2020) 9(9):891. doi: 10.3390/antiox9090891 PMC7555116 32962301

[6] Antonetti DA Silva PS Stitt AW. Current Understanding of the Molecular and Cellular Pathology of Diabetic Retinopathy. Nat Rev Endocrinol (2021) 17(4):195–206. doi: 10.1038/s41574-020-00451-4 33469209 PMC9053333

[7] Mustafi D Saraf SS Shang Q Olmos de Koo LC. New Developments in Angiography for the Diagnosis and Management of Diabetic Retinopathy. Diabetes Res Clin Pract (2020) 167:108361. doi: 10.1016/j.diabres.2020.108361 32745697

[8] Schreur V Larsen MB Sobrin L Bhavsar AR den Hollander AI Klevering BJ. Imaging Diabetic Retinal Disease: Clinical Imaging Requirements. Acta Ophthalmol (2022). doi: 10.1111/aos.15110 35142031

[9] Cai S Liu TYA. The Role of Ultra-Widefield Fundus Imaging and Fluorescein Angiography in Diagnosis and Treatment of Diabetic Retinopathy. Curr Diabetes Rep (2021) 21(9):30. doi: 10.1007/s11892-021-01398-0 34448948

[10] Pirola L Balcerczyk A Okabe J El-Osta A. Epigenetic Phenomena Linked to Diabetic Complications. Nat Rev Endocrinol (2010) 6(12):665–75. doi: 10.1038/nrendo.2010.188 21045787

[11] Salem TI Eldin NB Alhusseini NF Abdullah OA Ahmed NE. Expression Profile of Micrornas May Be Promising in Diagnosis of Proliferative Diabetic Retinopathy: An Egyptian Study. Int J Diabetes Developing Countries (2022). doi: 10.1007/s13410-022-01044-9

[12] Zhang J Cui C Xu H. Downregulation of Mir-145-5p Elevates Retinal Ganglion Cell Survival to Delay Diabetic Retinopathy Progress by Targeting Fgf5. Biosci Biotechnol Biochem (2019) 83(9):1655–62. doi: 10.1080/09168451.2019.1630251 31272285

[13] Greco M Chiefari E Accattato F Corigliano DM Arcidiacono B Mirabelli M. Microrna-1281 as a Novel Circulating Biomarker in Patients With Diabetic Retinopathy. Front Endocrinol (2020) 11:528. doi: 10.3389/fendo.2020.00528 PMC7417427 32849308

[14] Ye Z Li ZH He SZ. Mirna-1273g-3p Involvement in Development of Diabetic Retinopathy by Modulating the Autophagy-Lysosome Pathway. Med Sci Monit (2017) 23:5744–51. doi: 10.12659/msm.905336 PMC5724349 29197896

[15] Mori MA Ludwig RG Garcia-Martin R Brandao BB Kahn CR. Extracellular Mirnas: From Biomarkers to Mediators of Physiology and Disease. Cell Metab (2019) 30(4):656–73. doi: 10.1016/j.cmet.2019.07.011 PMC6774861 31447320

[16] Hill M Tran N. Mirna Interplay: Mechanisms and Consequences in Cancer. Dis Model Mech (2021) 14(4):dmm047662. doi: 10.1242/dmm.047662 PMC8077553 33973623

[17] Stevens MT Saunders BM. Targets and Regulation of Microrna-652-3p in Homoeostasis and Disease. J Mol Med (Berl) (2021) 99(6):755–69. doi: 10.1007/s00109-021-02060-8 33712860

[18] Yu H Liu H Chen X Wu N. Preliminary Study on Serum Microrna Expression Profile in Patients With Diabetic Retinopathy. Chin J Ophthalmol Med (2019) 9(04):246–51. doi: 10.3877/cma.j.issn.2095-2007.2019.04.009

[19] Chen X Rechavi O. Plant and Animal Small Rna Communications Between Cells and Organisms. Nat Rev Mol Cell Biol (2022) 23(3):185–203. doi: 10.1038/s41580-021-00425-y 34707241 PMC9208737

[20] Shaker OG Abdelaleem OO Mahmoud RH Abdelghaffar NK Ahmed TI Said OM. Diagnostic and Prognostic Role of Serum Mir-20b, Mir-17-3p, Hotair, and Malat1 in Diabetic Retinopathy. IUBMB Life (2019) 71(3):310–20. doi: 10.1002/iub.1970 30468285

[21] Xu YX Pu SD Li X Yu ZW Zhang YT Tong XW. Exosomal Ncrnas: Novel Therapeutic Target and Biomarker for Diabetic Complications. Pharmacol Res (2022) 178:106135. doi: 10.1016/j.phrs.2022.106135 35192956

[22] Whiting PF Rutjes AW Westwood ME Mallett S Deeks JJ Reitsma JB. Quadas-2: A Revised Tool for the Quality Assessment of Diagnostic Accuracy Studies. Ann Intern Med (2011) 155(8):529–36. doi: 10.7326/0003-4819-155-8-201110180-00009 22007046

[23] Higgins JP Thompson SG Deeks JJ Altman DG. Measuring Inconsistency in Meta-Analyses. BMJ (2003) 327(7414):557–60. doi: 10.1136/bmj.327.7414.557 PMC192859 12958120

[24] Jackson D White IR Thompson SG. Extending Dersimonian and Laird's Methodology to Perform Multivariate Random Effects Meta-Analyses. Stat Med (2010) 29(12):1282–97. doi: 10.1002/sim.3602 19408255

[25] Qing S Yuan S Yun C Hui H Mao P Wen F. Serum Mirna Biomarkers Serve as a Fingerprint for Proliferative Diabetic Retinopathy. Cell Physiol Biochem Int J Exp Cell Physiol Biochem Pharmacol (2014) 34(5):1733–40. doi: 10.1159/000366374 25427542

[26] Fu C. Expression of Mir-93 and Mir-21 in Patients With Diabetic Retinopathy and Its Clinical Values. Rec Adv Ophthalmol (2017) 37(12):1161–4. doi: 10.13389/j.cnki.rao.2017.0293

[27] Qin LL An MX Liu YL Xu HC Lu ZQ. Microrna-126: A Promising Novel Biomarker in Peripheral Blood for Diabetic Retinopathy. Int J Ophthalmol (2017) 10(4):530–4. doi: 10.18240/ijo.2017.04.05 PMC5406628 28503423

[28] Jiang Q Lyu XM Yuan Y Wang L. Plasma Mir-21 Expression: An Indicator for the Severity of Type 2 Diabetes With Diabetic Retinopathy. Biosci Rep (2017) 37(2):BSR20160589. doi: 10.1042/BSR20160589 PMC5469322 28108673

[29] Zou HL Wang Y Gang Q Zhang Y Sun Y. Plasma Level of Mir-93 Is Associated With Higher Risk to Develop Type 2 Diabetic Retinopathy. Graefe's Arch Clin Exp Ophthalmol Albrecht Von Graefes Archiv Fur Klin Und Experimentelle Ophthalmol (2017) 255(6):1159–66. doi: 10.1007/s00417-017-3638-5 28382439

[30] Sun J Cai R Gao Z Zhou T Yin W Hu H. Correlation of Plasma Microrna-21 Expression With Type 2 Diabetes Mellitus With Retinopathy. Guizhou Med J (2018) 42(12):1471–3. doi: CNKI:SUN:GZYI.0.2018-12-033

[31] Zheng H Yu J Tian N Wu J. Expression and Significances of Mir-126 and Vegf in Proliferative Diabetic Retinopathy. J Chin Physician (2018) 20(10):1477–81. doi: 10.3760/cma.j.issn.1008-1372.2018.10.010

[32] Liang Z Gao KP Wang YX Liu ZC Tian L Yang XZ. Rna Sequencing Identified Specific Circulating Mirna Biomarkers for Early Detection of Diabetes Retinopathy. Am J Physiol Endocrinol Metab (2018) 315(3):E374–e85. doi: 10.1152/ajpendo.00021.2018 29812988

[33] Dai B Dai Y. Clinical Value of Serum Mirna in Patients With Diabetic Retinopathy. Rec Adv Ophthalmol (2019) 39(08):780–4. doi: 10.13389/j.cnki.rao.2019.0178

[34] Ma Y Zhou LX Liu Y Wu W. Prediction of Retinopathy in Type 2 Diabetes Mellitus by Combined Detection of Microrna93 and Microrna21. Int Eye Sci (2019) 19(9):1550–3. doi: 10.3980/j.issn.1672-5123.2019.9.24

[35] Li Z Dong Y He C Pan X Liu D Yang J. Rna-Seq Revealed Novel Non-Proliferative Retinopathy Specific Circulating Mirnas in T2dm Patients. Front Genet (2019) 10:531. doi: 10.3389/fgene.2019.00531 31275351 PMC6593299

[36] Ji HH Yi QY Chen LS Wong LP Liu YF Xu GD. Circulating Mir-3197 and Mir-2116-5p as Novel Biomarkers for Diabetic Retinopathy. Clin Chim Acta (2019) 501:147–53. doi: 10.1016/j.cca.2019.10.036 31678272

[37] Lin Y Li Q Lin X. Expression and Clinical Significances of Mir-15 and Mir-29a in Serum of Patients With Diabetic Retinopathy. J Chin Physician (2020) 22(03):399–403. doi: 10.3760/cma.j.cn.431274-20181220-02388

[38] Liu L Wu B Ni L Zhang L Hu X Wang Z. Changes of Mir-15a,Mir-16 and Mir-20b in Patients With Type 2 Diabetes Mellitus and Their Relationship With Diabetic Retinopathy. Chin J Gen Pract (2020) 18(06):954–8. doi: 10.16766/j.cnki.issn.1674-4152.001401

[39] Yin C Lin X Sun Y Ji X. Dysregulation of Mir-210 Is Involved in the Development of Diabetic Retinopathy and Serves a Regulatory Role in Retinal Vascular Endothelial Cell Proliferation. Eur J Med Res (2020) 25(1):20. doi: 10.1186/s40001-020-00416-3 32498701 PMC7271497

[40] Wan Q Lei J Ma T. Risk Factors of Depression and Anxiety and Nursing Intervention in Diabetics During Perioperative Period. Acta Med Mediterr (2020) 36(5):3221–7. doi: 10.19193/0393-6384_2020_5_496

[41] Hu Q Luan L Liu Z. The Relationship Between Serum Mir-29c, Bfgf Levels and Diabetic Retinopathy. Chin J Atheroscl (2021) 29(12):1053–8. doi: 10.3969/j.issn.1007-3949.2021.12.008

[42] Liu Q Wang S Ding L Qin Y Maidina N. Diagnostic Value of Serum Mir-211 in Diabetic Retinopathy and Its Influencing Factors. Chin J Clin Lab Sci (2021) 39(06):445–8. doi: 10.13602/j.cnki.jcls.2021.06.10

[43] Sun Z Yang J. Correlation Between the Expression of Serum Mir⁃320 and Diabetic Retinopathy in Type 2 Diabetic Patients. Chin J Diabetes (2021) 29(05):344–8. doi: 10.3969/j.issn.10066187.2021.05.005

[44] Santovito D Toto L De Nardis V Marcantonio P D'Aloisio R Mastropasqua A. Plasma Microrna Signature Associated With Retinopathy in Patients With Type 2 Diabetes. Sci Rep (2021) 11(1):4136. doi: 10.1038/s41598-021-83047-w 33602976 PMC7892881

[45] Wang Z Zhang X Wang Y Xiao D. Dysregulation of Mir-374a Is Involved in the Progression of Diabetic Retinopathy and Regulates the Proliferation and Migration of Retinal Microvascular Endothelial Cells. Clin Exp Optometry (2021) 105:1–6. doi: 10.1080/08164622.2021.1913043 33941051

[46] Liu X Zhou Y Liu Y Wang Q Pan L. Microrna-425-5p Is Involved in the Development of Diabetic Retinopathy and Regulates the Proliferation and Migration of Retinal Microvascular Endothelial Cells. Ophthalmic Res (2022) 65(1):60–7. doi: 10.1159/000516906 34571504

[47] Glas AS Lijmer JG Prins MH Bonsel GJ Bossuyt PM. The Diagnostic Odds Ratio: A Single Indicator of Test Performance. J Clin Epidemiol (2003) 56(11):1129–35. doi: 10.1016/s0895-4356(03)00177-x 14615004

[48] Zhou H Peng C Huang DS Liu L Guan P. Microrna Expression Profiling Based on Microarray Approach in Human Diabetic Retinopathy: A Systematic Review and Meta-Analysis. DNA Cell Biol (2020) 39(3):441–50. doi: 10.1089/dna.2019.4942 32101049

[49] Stavast CJ Erkeland SJ. The Non-Canonical Aspects of Micrornas: Many Roads to Gene Regulation. Cells (2019) 8(11):1465. doi: 10.3390/cells8111465 PMC6912820 31752361

[50] Donati S Ciuffi S Brandi ML. Human Circulating Mirnas Real-Time Qrt-Pcr-Based Analysis: An Overview of Endogenous Reference Genes Used for Data Normalization. Int J Mol Sci (2019) 20(18):4353. doi: 10.3390/ijms20184353 PMC6769746 31491899

[51] Chen Q Qiu F Zhou K Matlock HG Takahashi Y Rajala RVS. Pathogenic Role of Microrna-21 in Diabetic Retinopathy Through Downregulation of Pparalpha. Diabetes (2017) 66(6):1671–82. doi: 10.2337/db16-1246 PMC5440012 28270521

[52] La Sala L Cattaneo M De Nigris V Pujadas G Testa R Bonfigli AR. Oscillating Glucose Induces Microrna-185 and Impairs an Efficient Antioxidant Response in Human Endothelial Cells. Cardiovasc Diabetol (2016) 15:71. doi: 10.1186/s12933-016-0390-9 27137793 PMC4852407

[53] La Sala L Mrakic-Sposta S Tagliabue E Prattichizzo F Micheloni S Sangalli E. Circulating Mirorna-21 Is an Early Predictor of Ros-Mediated Damage in Subjects With High Risk of Developing Diabetes and in Drug-Naive T2d. Cardiovasc Diabetol (2019) 18(1):18. doi: 10.1186/s12933-019-0824-2 30803440 PMC6388471

